# External validation of the TRISS, CRASH, and IMPACT prognostic models in severe traumatic brain injury in Japan

**DOI:** 10.1371/journal.pone.0221791

**Published:** 2019-08-26

**Authors:** Yukihiro Maeda, Rie Ichikawa, Jimpei Misawa, Akiko Shibuya, Teruyoshi Hishiki, Takeshi Maeda, Atsuo Yoshino, Yoshiaki Kondo

**Affiliations:** 1 Department of Health Care Services Management, Nihon University School of Medicine, Tokyo, Japan; 2 Department of Pediatrics and Child Health, Nihon University School of Medicine, Tokyo, Japan; 3 Department of Nursing, Toyama Prefectural University School of Nursing, Toyama, Japan; 4 Department of Information Science, Faculty of Science, Toho University, Chiba, Japan; 5 Department of Neurological Surgery, Nihon University School of Medicine, Tokyo, Japan; John Hunter Hospital and University of Newcastle, AUSTRALIA

## Abstract

In Japan, a range of patients with traumatic brain injury (TBI) has been recorded in a nationwide database (Japan Neurotrauma Data Bank; JNTDB). This study aimed to externally validate three international prediction models using JNTDB data: Trauma and Injury Severity Score (TRISS), Corticosteroid Randomization After Significant Head Injury (CRASH), and International Mission for Prognosis and Analysis of Clinical Trials in TBI (IMPACT). We also aimed to validate the applicability of these models in the Japanese population. Of 1,091 patients registered in the JNTDB from July 2009 to June 2011, we analyzed data for 635 patients. We examined factors associated with mortality in-hospital and unfavorable outcomes 6 months after TBI by applying the TRISS, CRASH, and IMPACT models. We also conducted an external validation of these models based on these data. The patients’ mean age was 60.1 ±21.1 years, and 342 were alive at the time of discharge (53.9%). Univariate analysis revealed eight major risk factors for mortality in-hospital: age, Glasgow Coma Scale (GCS), Injury Severity Score (ISS), systolic blood pressure, heart rate, mydriasis, acute epidural hematoma (AEDH), and traumatic subarachnoid hemorrhage. A similar analysis identified five risk factors for unfavorable outcomes at 6 months: age, GCS, ISS, mydriasis, and AEDH. For mortality in-hospital, the TRISS had a satisfactory area under the curve value (0.75). For unfavorable outcomes at 6 months, the CRASH (basic and computed tomography) and IMPACT (core and core extended) models had satisfactory area under the curve values (0.86, 0.86, 0.81, and 0.85, respectively). The TRISS, CRASH, and IMPACT models were suitable for application to the JNTDB population, indicating these models had high value in Japanese patients with neurotrauma.

## Introduction

Traumatic head injury is a major cause of death [1]. Practitioners are requested to identify patients with such injuries who will survive after arrival at the emergency department, and start appropriate medical practice immediately. For this reason, many attempts to establish appropriate prediction models have been conducted worldwide [2–14]. Available models include the Abbreviated Injury Scale developed by the Association for the Advancement of Automobile Medicine [2] and the Injury Severity Score (ISS) for evaluating emergency care for multiple injuries [3]. The Traumatic Coma Data Bank, which was a multicenter epidemiological study of traumatic head injury conducted in the USA [4], led to the evaluation and classification of computed tomography findings for prediction of prognosis [5, 6].

The Trauma and Injury Severity Score (TRISS) model [7] was developed in 1987, and has been used as a tool for predicting survival [8]. The TRISS includes the ISS for anatomical severity [3] and Revised Trauma Score for physiologic reserve. The TRISS also covers the Glasgow Coma Scale (GCS), systolic blood pressure (SBP), respiratory rate (RR), and age [7]. The TRISS method was later modified for intubated patients, which improved the prediction accuracy [9]. The modified Kampala [10], Trauma Mortality Prediction Model [11], and modified McPeek [12] were further suggested as models that improved prediction accuracy.

Recently, two new prediction models have been proposed and discussed worldwide: the Corticosteroid Randomization After Significant Head Injury (CRASH) and the International Mission for Prognosis and Analysis of Clinical Trials in Traumatic Brain Injury (IMPACT) [13]. The National Neuroscience Institute (NNI) in Singapore also reported new prediction models (NNI clinical and NNI+) following a cohort study [14].

The Japan Neurotrauma Data Bank (JNTDB) was founded in 1998, and is an authorized nationwide database for epidemiological studies of traumatic brain injury (TBI) [15]. The JNTDB has conducted three separate series of studies, with Project 2009 (conducted from 2009–2011) being the most recent open database available. Although the JNTDB is well established, there has been no comprehensive report on the availability of effective prediction models for death and prognosis following TBI in Japan, including the TRISS, CRASH, and IMPACT models.

The present study aimed to apply these international prediction models to JNTDB data and externally evaluate these models, thereby establishing the appropriateness of the models in the Japanese population.

## Methods

The dataset used in this study was drawn from the JNTDB [15]. The JNTDB started collecting data in 1998 To date, three studies have drawn on these data, the most recent being Project 2009, in which information for patients from 22 registered institutions was collected for 2 years (from 1 July 2009 to 30 June 2011), as in S1 Table. The JNTDB included patients of any age, but excluded patients with cardiopulmonary arrest on arrival (CPAOA) not suspected of being attributable to TBI. All patients with a GCS score ≤8 on admission or during follow-up were included. Patients with a GCS score >8 who had undergone craniotomy (chronic subdural hematoma excluded; burr holes included) were also included.

The inclusion and exclusion criteria for patients in the present study are presented in Table 1. Project 2009 included 1,091 patients. Of those patients, 325 patients with a GCS score >8 were excluded from the present study. After exclusion of 36 patients younger than 16 years, one patient with unknown outcome at discharge, and 94 patients with CPAOA, data for 635 patients remained for analysis in this study. Of these patients, 265 (41.7%) had been injured in motor vehicle accidents and 309 (48.7%) by falls. Among the 635 cases, 293 cases died in hospital. For unfavorable outcome at 6 months, an additional 11 cases died after discharge, meaning that 304 of the 635 cases died.

**Table 1:** Inclusion and exclusion criteria for patients in the present study.

Inclusion criteria
(1) Age equal to or older than 16 years
(2) GCS score ≤8 on admission
Exclusion criteria
(3) CPAOA

GCS: Glasgow Coma Scale; CPAOA: cardiopulmonary arrest on arrival.

Permission to analyze JNTDB data was obtained from the Japan Society of Neurotraumatology before starting this study. This study was approved by the Institutional Ethics Review Board of Nihon University School of Medicine (notice number 26–9). The requirement for patient consent was waived because only anonymized data were used.

### Statistical analysis

Univariate and multivariate analyses were performed for all factors recorded in the JNTDB. These factors included age, sex, ISS, GCS, SBP, heart rate, RR, Revised Trauma Score, Japan Coma Scale, body temperature, serum glucose (SG), mydriasis, cause of injury, physical examination findings, imaging findings, and outcome. For prediction of outcome at discharge, the states of “deceased” and “alive” were numerically defined as 0 and 1, respectively. Outcomes 6 months after TBI were assessed based on the Glasgow Outcome Scale. Outcomes were grouped into two categories: favorable outcome (good recovery and moderate recovery); and unfavorable outcome (severely disabled, vegetative states, and death).

The area under the receiver operating characteristic curve (AUC) was calculated for each model to enable comparison of data discrimination properties. Statistical analyses were performed with JMP version 10.0 (SAS Institute, Cary, NC, USA) and EZR version 1.37 (R Foundation for Statistical Computing, Vienna, Austria, version 3.4.1) [16]. P-values less than 0.05 were considered statistically significant.

## Results

Demographic and examined factors for patients in univariate analyses are summarized in Table 2. The mean age of patients with recorded mortality in hospital (M-IH) was 60.0 ± 21.1 years (range 16–98 years), and 69.7% were men. Logistic analysis of the three SBP groups showed statistically significant differences among the groups (p<0.0001, N = 627), indicating that both higher and lower SBP were related to higher mortality. Low heart rate also appeared to be related to higher mortality. No special tendency in terms of mortality was observed in relation to RR, but high SG and the presence of mydriasis were risk factors for mortality. In addition, the presence of acute subdural hematoma, intracerebral hemorrhage, and traumatic subarachnoid hemorrhage were risk factors for mortality, whereas the presence of acute epidural hematoma (AEDH) tended to reduce mortality. There appeared to be no significant relationship between cerebral contusion and mortality.

**Table 2:** 

	Mortality in hospital					Unfavorable outcome at 6 months				
Factor	No	Yes	N	VD	p-value	Favorable	Unfavorable	N	VD	p-value
	n (%)	n (%)		(%)		n (%)	n (%)		(%)	
Total	342 (53.9)	293 (46.1)	635	100		116 (22.7)	396 (77.3)	512	100	
Sex			635	100	0.0584			512	100	0.0116
Male	249 (56.3)	193 (43.7)				92 (25.6)	267 (74.4)			
Female	93 (48.2)	100 (51.8)				24 (15.7)	129 (84.3)			
Age, years			635	100				512	100	
16–54	137 (66.8)	68 (33.2)				81 (46.3)	94 (53.7)			
55–74	123 (52.6)	111 (47.4)				28 (15.7)	150 (84.3)			
≥75	82 (41.8)	114 (58.2)				7 (4.4)	152 (95.6)			
GCS score			635	100				512	100	<.0001
3	53 (31.9)	113 (68.1)				17 (11.6)	130 (88.4)			
4	45 (40.5)	66 (59.5)				8 (8.7)	84 (91.3)			
5	25 (56.8)	19 (43.2)				6 (17.1)	29 (82.7)			
6	67 (54.9)	55 (45.1)				28 (27.7)	73 (72.3)			
7	100 (78.7)	27 (21.3)				36 (39.6)	55 (60.4)			
8	52 (80.0)	13 (25.0)				21 (45.7)	25 (54.4)			
ISS			635	100	<.0001			512	100	<.0001
1–24	118 (81.9)	26 (18.1)				48 (48.5)	51 (51.5)			
25–75	224 (45.6)	267 (54.4)				68 (16.5)	345 (83.5)			
SBP, mmHg			627	98.7				505	98.6	<.0001
1–89	15 (31.9)	32 (68.1)				2 (5.4)	35 (94.6)			
90–180	286 (61.1)	182 (38.9)				107 (28.7)	266 (71.3)			
≥181	39 (34.8)	73 (65.2)				7 (7.4)	88 (92.6)			
HR, /min			634	99.8	0.0048			512	100	0.5266
<60	21 (36,2)	37 (63.8)				10 (19.2)	42 (80.1)			
≥60	320 (55.6)	256 (44.4)				106 (23.0)	354 (77.0)			
RR,/min			607	95.6				495	96.7	0.2751
>29	40 (54.1)	34 (46.0)				18 (27.7)	47 (72.3)			
10–29	282 (54.9)	232 (45.1)				93 (22.6)	319 (77.4)			
6–9	0 (0.0)	1 (100.0)				0 (0.0)	1 (100.0)			
1–5	0 (0.0)	1 (100.0)				0 (0.0)	1 (100.0)			
0	4 (25.5)	13 (76.5)				1 (6.3)	15 (93.8)			
SG, mg/dL			624	98.3	<.0001			507	99	<.0001
<200	257 (61.9)	158 (38.1)				99 (30.0)	231 (70.0)			
≥200	78 (37.3)	131 (62.7)				17 (9.6)	160 (90.4)			
Mydriasis			634	99.8	<.0001			511	99.8	<.0001
Present	123 (36.7)	212 (63.3)				33 (11.3)	259 (88.7)			
Absent	218 (72.9)	81 (27.1)				82 (37.4)	137 (62.6)			
AEDH			635	100	<.0001			512	100	0.0007
Present	45 (81.8)	10 (18.2)				18 (46.2)	21 (53.9)			
Absent	297 (51.2)	283 (48.8)				98 (20.7)	375 (79.3)			
ASDH			635	100	<.0001			512	100	<.0001
Present	161 (46.0)	189 (54.0)				45 (15.9)	238 (84.1)			
Absent	181 (63.5)	104 (36.5)				71 (31.0)	158 (69.0)			
ICH			635	100	0.0721			512	100	0.1054
Present	17 (40.5)	25 (59.5)				5 (12.8)	34 (87.2)			
Absent	325 (54.8)	268 (45.2)				111 (23.5)	362 (76.5)			
CC			635	100	0.0555			512	100	0.4659
Present	74 (61.7)	46 (38.3)				24 (25.5)	70 (74.5)			
Absent	268 (52.0)	247 (48.0)				92 (22.0)	326 (78.0)			
tSAH			628	98.9	0.0741			509	99.4	0.5988
Present	241 (51.6)	227 (48.4)				86 (22.1)	304 (78.0)			
Absent	95 (59.8)	64 (40.3)				29 (24.4)	90 (75.6)			

AEDH: acute epidural hematoma; ASDH: acute subdural hematoma; CC: cerebral contusion; GCS: Glasgow Coma Scale; HR: heart rate; ICH: intracerebral hemorrhage; ISS: Injury Severity Score; N: number of patients; RR: respiratory rate; SBP: systolic blood pressure; SG: serum glucose; tSAH: traumatic subarachnoid hemorrhage; VD: valid data.

In total, 512 patients were included in the analysis for prediction of unfavorable outcome 6 months after TBI (UO-6M), 70.1% of which were men. The GCS was lower in patients with unfavorable outcomes, whereas the ISS was higher. Both low and high SBP tended to be related to poorer outcomes, but there was no obvious relationship between heart rate or RR and outcome. Higher SG may also be a risk factor for an unfavorable outcome. The presence of mydriasis and acute subdural hematoma appeared to be related to poorer outcomes, whereas there was no clear risk associated with the presence of intracerebral hemorrhage, cerebral contusion and traumatic subarachnoid hemorrhage. In contrast, the presence of AEDH indicated better outcomes for patients.

AUC analyses were performed to determine the efficacy of the TRISS, CRASH, and IMPACT models (Table 3). Cases with unavailable data for some factors were included in the analyses of 635 patients for M-IH and 512 patients UO-6M. As a result of eliminating those patients for the validation of each prediction model, the number of patients varied among the prediction models (Table 3). To compare the accuracy of the prediction models for M-IH, data for 600 patients were examined using the TRISS model, and showed an AUC of 0.75. For prediction of UO-6M, data were examined using CRASH basic (511 patients), CRASH computed tomography (504 patients), IMPACT core (511 patients), and IMPACT extended (450 patients). The IMPACT lab model could not be applied in this study because of the lack of blood hemoglobin data in the JNTDB. For UO-6M, the AUCs for the CRASH basic, CRASH computed tomography, IMPACT core, and IMPACT extended models were 0.86, 0.86, 0.81, and 0.85, respectively. The AUCs for the CRASH models tended to be higher than those for the IMPACT model.

**Table 3:** Area under the receiver operating characteristic curves for the TRISS, CRASH, and IMPACT models for external validation using Japan Neurotrauma Data Bank data.

Regression model	Factors used (n)	AUC	95% CI	N
Mortality in hospital				
TRISS	5	0.75	0.72–0.79	600
Unfavorable outcome at 6 months				
CRASH				
basic	4	0.86	0.82–0.90	511
CT	9	0.86	0.82–0.89	504
IMPACT				
core	3	0.81	0.77–0.85	511
extended	8	0.85	0.80–0.89	450
lab*	10	-	-	-

AUC: area under the receiver operating characteristic curve; CI: confidence interval; TRISS: Trauma and Injury Severity Score; CRASH: Corticosteroid Randomization After Significant Head Injury; IMPACT: International Mission for Prognosis and Analysis of Clinical Trials in Traumatic Brain Injury; CT: computed tomography.

*Prediction could not be performed because of lack of blood hemoglobin data in the Japan Neurotrauma Data Bank.

## Discussion

The major focus of the present study was the applicability of currently used international prediction models for patients with severe TBI in Japan. Although the TRISS model has often been applied for the Japanese population, there have been no evaluations of the applicability of other international models such as the CRASH and IMPACT models. This highlights a lack of information about sophisticated standards for predicting UO-6M in Japan. We therefore attempted to externally evaluate the three international prediction models for UO-6M based on JNTDB data.

A major concern was if the prediction models were applicable to patients severe TBI. Sophisticated predictive models should require minimal routinely-measured factors, which may save clinical time and resources. In the JNTDB, RR and SG were the prediction factors most commonly missing. Of the 635 patients included in the present study, RR and SG were not recorded for 4.4% and 1.7% of patients, respectively. Because RR is needed for calculating TRISS scores, TRISS scores could only be calculated for 600 patients (94.5%). Despite the TRISS model having limited applicability to JNTDB data, the AUC for the TRISS was 0.75, indicating this model can be used for the Japanese population. However, no authorized Japanese prediction models are available to date.

The applicability of the CRASH and IMPACT models for predicting UO-6M for **512** patients is summarized in Table 3. The prediction scores for the CRASH basic and computed tomography models could be calculated for 511 (99.8%) and 450 (87.9%) patients, respectively. Core and extended IMPACT scores could be calculated for 511 (99.8%) and 450 (87.9%) patients, respectively.

Internationally, a number of studies have examined the usefulness of the TRISS, CRASH, and IMPACT models. In 2016, Sun et al. summarized the performance of the IMPACT models as determined by external validation [17]. They compared six studies that had investigated prediction of mortality or unfavorable outcomes [14, 18–22]. The AUC ranges for mortality prediction using the IMPACT models were 0.65–0.85 (core), 0.69–0.88 (extended), and (0.69–0.90) lab. For unfavorable outcome prediction, the ranges were 0.66–0.84 (core), 0.71–0.88 (extended), and 0.70–0.87 (lab). In the JNTDB, the AUCs for the core and extended IMPACT models were 0.81 and 0.85, respectively. These AUCs suggest the IMPACT models have a comparable fit with patients in Japan, although these scores can be calculated for fewer patients.

Han et al. reported that the new NNI models performed better than CRASH and IMPACT models, as indicated by a lower Akaike information criterion and greater AUC [14]. We were unable to analyze their models using JNTDB data because of insufficient information on the model’s structure. Future studies are needed to determine the applicability and limitations of those models for JNTDB data.

Brennan et al. recently proposed a simplified prediction model for TBI prognosis called the GCS-Pupils score (GCS-P), which was derived from the CRASH and IMPACT databases [23]. The GCS-P combines the GCS score and state of the pupils with ranges of 3–15 and 0–2, respectively (the overall GCS-P has a range of 1–15). The simplicity of the model makes it convenient for clinical staff; however, the loss of accuracy in prediction is a potential disadvantage of the GCS-P. To clarify the value of the GCS-P, further study is necessary using JNTDB data.

We could not determine why the presence of AEDH was a better prognostic factor for UO-6M in our multivariate analysis. In patients with AEDH, the complication of acute subdural hematoma was significantly less frequent (p<0.0001). Such a difference in these patients’ pathological state might have influenced the prognosis of patients with AEDH. Further studies are necessary to clarify this issue.

The present study had several major limitations. First, the obtained data were limited to patients who had been admitted to the 22 participating institutions. Of note, all institutions that participated in JNTDB Project 2009 were either university hospitals or central medical centers. It is possible that the characteristics of patients with TBI presenting to small hospitals differ from those of patients presenting to major institutions. Second, we were unable to compare the accuracy of prediction for the IMPACT lab, NNI, and NNI+ models because we were unable to obtain the prediction equations for these models and therefore could not assess them using JNTDB Project 2009 data. It is possible that these models would have better AUCs in our dataset. Third, the JNTDB dataset used for the present analysis was not contemporary because of limitations in access to raw data for our research purposes. The overall mortality and outcomes after TBI in Japan are likely to have been improved following changes in resuscitation techniques (e.g., volume resuscitation) since 2009 [24]. We intend to reevaluate these prediction models using contemporary data from the next available database in the near future. Fourth, we could externally evaluate three international prediction models only for patients with severe TBI because of the limitation of bias in the inclusion criteria for the JNTDB dataset. The JNTDB Project 2009 included three categories of patients aged 16 years or older: patients with a GCS score of ≤8, patients with a GCS score of ≤8 during follow up, and patients with a GCS score >8 who had undergone craniotomy. The major bias for inclusion of patients for the present study lies with the latter two groups because these groups were not defined in the three prediction models. Inclusion of these groups might have led to partial inclusion of patients with a GCS score >8 on admission, leading to incorrect validation of the models. For perfect validation of three models using JNTDB data, it would be necessary to include all patients with a GCS >8. Nevertheless, our results clearly indicate that all three models were appropriate for prediction of prognosis in those with severe TBI. It should also be noted that we did not perform a power analysis to evaluate the reliability of the JNTDB. A power analysis would have allowed estimation of the quality of the JNTDB data. However, the JNTDB is the only database for TBI in Japan, and we had no other options against which to compare the appropriateness of our analyses in the present study. Power analysis will be of particular value when we analyze the next JNTDB project in comparison with the results of the present study.

In addition to our previous approach to establish the prediction model for severe head injury in Japanese children [25], the present external validation of the TRISS, CRASH, and IMPACT models has clarified their value for use in Japan.

## Conclusion

A systematic external validation of the TRISS, CRASH, and IMPACT prediction models revealed these models have convincing values for prediction of outcomes for Japanese patients with severe TBI. These models should be widely applied in institutions admitting patients with severe TBI in Japan.

## Supporting information

S1 Table Details of cases for each hospital. (PDF)
