# Supplementary material for: External validation of the TRISS, CRASH, and IMPACT prognostic models in severe traumatic brain injury in Japan
Source: PLoS One. 2019 Aug 26;14(8):e0221791. doi: 10.1371/journal.pone.0221791 (PMC6709937; doi:10.1371/journal.pone.0221791)
Supplement: S1 Table — (PDF) [file pone.0221791.s001.pdf]

Hospital Name	Number of cases	mortality in-hospital (n)	mortality in-hospital: Death	mortality in-hospital: Alive	Glasgow Outcome Scale: 5 Good recovery	Glasgow Outcome Scale: 4 Moderate disability	Glasgow Outcome Scale: 3 Severe disability	Glasgow Outcome Scale: 2 Vegetative state	Glasgow Outcome Scale: 1 Dead	outcomes 6 months after TBI (n)	outcomes 6 months after TBI: 5 Good recovery	outcomes 6 months after TBI: 4 Moderate	outcomes 6 months after TBI: 3 Severe disability	outcomes 6 months after TBI: 2 Vegetative	outcomes 6 months after TBI: 1 Dead	outcomes 6 months after TBI: unfavorable	outcomes 6 months after TBI: favorable	Age (n)	Age (mean)	Age (SD)	Age (min)	Age (25percentile)	Age (median)	Age (75percentile)	Age (max)	Age: 16-54	Age: 55-74	Age: ≥75	Sex: Female	Sex: Male	GCS:3	GCS:4	GCS:5	GCS:6	GCS:7	GCS:8	GCS:9	GCS:10	GCS:11	GCS:12
A	78	78	34	44	2	3	25	14	34	48	3	3	5	3	34	42	6	78	59.4	19.7	19	42.8	62.5	76.0	87	25	31	22	26	52	19	14	4	12	20	9	0	0	0	0
B	67	67	24	43	8	13	12	10	24	38	4	9	0	1	24	25	13	67	61.8	20.3	20	46.0	62.0	80.0	92	25	19	23	22	45	19	8	2	18	14	6	0	0	0	0
C	61	61	30	31	5	6	12	8	30	60	9	2	12	7	30	49	11	61	62.9	20.3	16	47.0	69.0	78.5	91	20	19	22	16	45	24	3	2	18	11	3	0	0	0	0
D	61	61	34	27	5	5	10	7	34	61	9	6	5	7	34	46	15	61	57.4	22.8	17	38.5	62.0	75.5	95	23	22	16	12	49	20	18	7	7	8	1	0	0	0	0
E	39	39	21	18	0	4	9	5	21	39	4	1	6	3	25	34	5	39	66.1	21.0	16	56.0	69.0	81.0	97	8	14	17	13	26	9	9	2	7	9	3	0	0	0	0
F	46	46	21	25	1	7	8	9	21	44	5	5	4	5	25	34	10	46	59.2	24.1	16	39.8	68.5	78.0	98	16	13	17	15	31	10	10	3	10	10	3	0	0	0	0
G	35	35	15	20	7	6	5	2	15	34	10	3	4	1	16	21	13	35	59.8	17.9	18	49.0	63.0	74.0	85	10	18	7	5	30	10	2	1	7	9	6	0	0	0	0
H	38	38	20	18	3	4	6	5	20	21	0	1	0	0	20	20	1	38	60.9	21.3	20	40.5	68.0	78.3	94	12	12	14	16	22	11	2	2	7	9	7	0	0	0	0
J	28	28	14	14	6	3	3	2	14	20	4	1	0	1	14	15	5	28	46.3	22.6	17	22.5	54.5	62.0	84	14	10	4	6	22	7	3	4	4	6	4	0	0	0	0
K	29	29	17	12	2	0	7	3	17	26	3	0	2	3	18	23	3	29	68.7	15.6	20	61.5	71.0	77.5	89	4	13	12	14	15	7	7	2	6	4	3	0	0	0	0
L	25	25	17	8	1	4	0	3	17	24	2	3	1	0	18	19	5	25	58.6	25.2	17	30.5	69.0	79.5	95	9	6	10	8	17	6	7	1	2	4	5	0	0	0	0
M	22	22	7	15	2	8	3	2	7	22	6	5	2	2	7	11	11	22	53.3	20.5	16	35.0	62.0	67.5	90	10	10	2	6	16	5	3	2	6	4	2	0	0	0	0
N	26	26	10	16	1	4	6	5	10	25	5	1	7	2	10	19	6	26	57.7	20.4	16	39.8	66.5	73.5	82	9	11	6	6	20	4	6	4	4	6	2	0	0	0	0
P	22	22	9	13	1	4	6	2	9	11	1	0	0	1	9	10	1	22	59.4	17.6	19	54.3	62.0	71.3	95	5	13	4	6	16	3	5	1	7	5	1	0	0	0	0
Q	10	10	0	10	2	1	7	0	0	1	1	0	0	0	0	0	1	10	61.6	19.3	17	56.0	63.0	72.3	88	2	6	2	3	7	2	3	2	1	1	1	0	0	0	0
R	15	15	4	11	2	3	3	3	4	6	0	1	1	0	4	5	1	15	58.5	27.0	17	20.0	64.0	80.0	93	5	4	6	8	7	2	3	2	2	4	2	0	0	0	0
S	14	14	4	10	0	4	6	0	4	14	1	4	5	0	4	9	5	14	61.4	19.4	27	44.5	62.5	78.0	88	4	6	4	5	9	3	4	1	0	1	5	0	0	0	0
T	4	4	3	1	0	1	0	0	3	4	1	0	0	0	3	3	1	4	59.0	25.7	21	32.3	69.5	75.3	76	1	2	1	1	3	2	1	0	1	0	0	0	0	0	0
U	5	5	2	3	2	0	1	0	2	5	2	0	1	0	2	3	2	5	48.2	22.9	29	29.5	40.0	71.0	83	3	1	1	2	3	1	1	0	1	0	2	0	0	0	0
V	5	5	4	1	0	0	0	1	4	4	0	0	0	0	4	4	0	5	84.0	7.7	76	76.5	84.0	91.5	94	0	0	5	2	3	1	1	1	2	0	0	0	0	0	0
W	3	3	1	2	0	1	0	1	1	3	0	1	0	1	1	2	1	3	67.7	10.0	58	58.0	67.0	78.0	78	0	2	1	1	2	0	0	1	0	2	0	0	0	0	0
X	2	2	2	0	0	0	0	0	2	2	0	0	0	0	2	2	0	2	71.0	0.0	71	71.0	71.0	71.0	71	0	2	0	0	2	1	1	0	0	0	0	0	0	0	0

Hospital Name	GCS:13	GCS:14	GCS:15	GCS-E:1	GCS-E:2	GCS-E:3	GCS-E:4	GCS-V:1	GCS-V:2	GCS-V:3	GCS-V:4	GCS-V:5	GCS-M:1	GCS-M:2	GCS-M:3	GCS-M:4	GCS-M:5	GCS-M:6	ISS:1	ISS:2	ISS:3	ISS:4	ISS:5	ISS:6	ISS:9	ISS:10	ISS:11	ISS:12	ISS:13	ISS:14	ISS:16	ISS:17	ISS:18	ISS:20	ISS:21	ISS:22	ISS:24	ISS:25	ISS:26
A	0	0	0	73	4	1	0	67	11	0	0	0	19	14	5	19	20	1	0	0	0	1	0	0	0	1	0	0	1	1	2	2	1	0	1	0	1	20	8
B	0	0	0	64	2	0	1	56	11	0	0	0	21	8	2	20	16	0	0	0	0	0	0	0	0	0	0	0	0	1	4	1	0	1	0	0	0	33	2
C	0	0	0	59	2	0	0	52	8	1	0	0	25	4	0	24	8	0	0	0	0	0	0	0	0	1	0	0	3	1	9	1	0	3	1	0	0	14	1
D	0	0	0	57	4	0	0	59	2	0	0	0	21	17	7	11	5	0	0	0	0	0	0	0	0	0	0	0	0	0	4	3	0	0	1	0	2	20	1
E	0	0	0	37	2	0	0	36	3	0	0	0	9	9	2	9	10	0	0	0	0	0	0	2	1	0	1	0	0	2	1	1	3	0	2	0	1	4	5
F	0	0	0	42	3	1	0	42	4	0	0	0	11	9	3	15	8	0	1	0	0	1	0	0	1	0	0	0	0	0	10	1	0	2	0	0	0	21	1
G	0	0	0	34	0	0	1	28	7	0	0	0	10	4	0	7	14	0	0	0	0	0	0	0	2	0	0	0	1	0	4	1	0	0	1	0	0	9	7
H	0	0	0	35	2	1	0	28	9	1	0	0	11	2	3	13	9	0	1	0	1	0	0	0	1	1	0	0	0	0	5	1	0	0	2	0	0	11	0
J	0	0	0	25	3	0	0	25	3	0	0	0	7	3	5	6	5	2	0	0	0	0	0	0	0	1	0	0	1	0	1	0	0	0	0	1	0	3	7
K	0	0	0	24	4	1	0	27	2	0	0	0	8	7	1	9	4	0	0	0	0	0	0	0	1	0	0	0	0	0	1	2	0	0	0	0	0	12	3
L	0	0	0	23	0	2	0	17	7	1	0	0	6	7	2	8	2	0	0	0	0	0	0	0	0	0	0	0	0	0	0	0	0	0	1	0	0	14	1
M	0	0	0	19	2	0	1	18	4	0	0	0	8	1	2	7	4	0	0	0	0	0	0	0	1	0	0	0	0	0	3	0	1	0	0	0	0	4	4
N	0	0	0	23	2	1	0	19	7	0	0	0	5	6	3	11	1	0	0	0	0	0	0	0	0	0	0	0	0	0	0	0	1	0	0	0	0	2	10
P	0	0	0	22	0	0	0	19	3	0	0	0	4	4	1	8	5	0	0	0	1	0	0	2	0	0	0	0	0	0	0	0	0	0	0	0	0	16	1
Q	0	0	0	9	1	0	0	8	2	0	0	0	2	3	3	1	1	0	2	0	2	0	0	0	1	0	1	0	0	0	4	0	0	0	0	0	0	0	0
R	0	0	0	13	1	1	0	11	3	0	1	0	3	3	3	3	3	0	0	0	0	0	0	0	2	0	0	0	1	1	2	1	1	1	0	0	0	4	0
S	0	0	0	10	3	0	1	9	5	0	0	0	3	5	1	3	2	0	0	0	0	0	0	0	0	0	0	0	0	0	2	0	0	0	0	0	0	5	3
T	0	0	0	4	0	0	0	4	0	0	0	0	2	1	0	1	0	0	1	1	0	0	0	0	0	0	0	0	0	0	1	0	1	0	0	0	0	0	0
U	0	0	0	3	2	0	0	5	0	0	0	0	1	1	0	1	2	0	0	0	0	0	0	0	0	0	0	0	0	0	0	1	0	0	0	0	0	1	1
V	0	0	0	5	0	0	0	5	0	0	0	0	1	1	1	2	0	0	0	0	0	0	0	0	0	0	0	0	0	0	0	1	0	0	0	0	0	1	0
W	0	0	0	3	0	0	0	3	0	0	0	0	0	0	1	0	2	0	0	1	0	0	0	0	0	0	0	0	0	0	0	0	0	0	0	0	0	0	0
X	0	0	0	2	0	0	0	1	1	0	0	0	2	0	0	0	0	0	0	0	0	0	0	0	0	0	0	0	0	0	0	0	0	0	0	0	0	0	0

Hospital Name	ISS:27	ISS:29	ISS:30	ISS:32	ISS:33	ISS:34	ISS:35	ISS:36	ISS:37	ISS:38	ISS:41	ISS:42	ISS:43	ISS:45	ISS:48	ISS:50	ISS:54	ISS:57	ISS:59	ISS:66	ISS:75	SBP (n)	SBP (mean)	SBP (SD)	SBP (min)	SBP (25percentile)	SBP (median)	SBP (75percentile)	SBP (max)	SBP: 1-89	SBP: 90-180	SBP: >180	Heart rate (n)	Heart rate (mean)	Heart rate (SD)	Heart rate (min)	Heart rate (25percentile)	Heart rate (median)	Heart rate (75percentile)
A	0	6	1	0	2	9	3	3	0	4	3	1	1	4	1	1	0	0	0	0	0	75	131.0	43.9	40	100.0	130.0	160.0	220	11	55	9	78	100.9	29.4	0	80.0	98.0	120.0
B	0	5	0	0	0	3	0	1	0	2	4	0	1	3	1	2	1	0	0	2	0	67	143.2	41.7	54	117.0	142.0	171.0	262	6	51	10	67	91.2	23.9	47	78.0	90.0	105.0
C	1	5	1	0	2	7	0	1	0	4	2	0	1	0	0	1	0	0	1	1	0	59	146.4	34.6	65	125.0	147.0	163.0	242	2	50	7	61	96.0	28.3	50	75.5	90.0	120.5
D	0	5	1	0	1	2	1	2	0	2	2	2	0	3	0	3	0	1	0	1	4	61	143.1	48.1	44	110.0	143.0	166.0	341	6	46	9	61	97.1	27.6	42	78.0	95.0	118.0
E	4	1	0	0	0	1	1	0	0	0	0	0	0	0	0	0	0	0	0	0	9	39	156.6	34.9	67	132.0	158.0	185.0	218	1	27	11	39	86.2	25.8	50	65.0	80.0	104.0
F	0	0	1	0	0	2	0	0	0	1	2	0	1	0	0	1	0	0	0	0	0	45	169.1	40.6	87	143.5	161.0	197.0	280	1	30	14	46	85.6	25.3	39	72.0	83.0	98.5
G	0	4	1	0	2	0	0	0	0	3	0	0	0	0	0	0	0	0	0	0	0	35	148.7	37.5	64	112.0	152.0	180.0	210	1	28	6	35	92.1	26.1	45	76.0	90.0	102.0
H	3	1	0	0	1	0	0	0	1	0	1	0	2	1	0	0	0	0	0	0	5	37	144.5	37.5	57	115.5	144.0	169.5	245	1	31	5	38	87.6	29.1	0	73.8	87.0	101.3
J	4	0	0	0	0	0	1	0	0	3	0	2	0	0	0	3	0	0	0	0	1	28	147.2	36.4	73	117.5	149.5	173.8	223	1	22	5	28	94.5	26.7	56	71.5	92.0	115.0
K	2	0	2	0	0	0	1	0	0	1	1	0	1	0	0	0	0	0	0	1	1	29	140.9	40.8	42	117.5	139.0	173.0	199	3	20	6	29	89.9	20.3	62	73.5	89.0	100.5
L	1	0	0	0	1	0	0	0	0	0	0	1	2	0	0	1	0	0	2	0	1	25	134.8	38.0	72	119.5	131.0	161.0	211	5	17	3	25	92.8	24.5	58	75.5	86.0	110.0
M	1	3	0	1	0	1	1	0	0	0	1	1	0	0	0	0	0	0	0	0	0	22	149.6	42.3	70	111.5	143.0	190.0	220	1	14	7	22	80.9	21.2	58	64.0	73.5	94.8
N	4	1	1	0	1	0	1	0	0	1	0	0	0	0	0	4	0	0	0	0	0	26	150.3	45.2	51	117.5	150.0	187.0	240	2	17	7	26	89.0	24.8	42	70.5	84.0	110.0
P	0	0	0	0	0	0	1	0	0	0	0	0	0	1	0	0	0	0	0	0	0	22	159.9	32.9	103	137.0	163.0	185.8	220	0	16	6	22	70.2	12.5	50	60.0	69.0	80.0
Q	0	0	0	0	0	0	0	0	0	0	0	0	0	0	0	0	0	0	0	0	0	10	146.4	50.5	68	102.3	150.5	182.5	229	2	6	2	10	86.9	19.9	60	74.5	84.0	94.0
R	0	1	0	0	0	0	0	1	0	0	0	0	0	0	0	0	0	0	0	0	0	14	128.3	31.6	72	94.3	134.5	152.3	170	3	11	0	14	89.7	25.7	54	70.3	84.5	111.3
S	3	0	1	0	0	0	0	0	0	0	0	0	0	0	0	0	0	0	0	0	0	14	144.1	35.6	82	115.0	141.0	174.0	210	1	11	2	14	84.7	32.4	40	64.5	75.0	92.8
T	0	0	0	0	0	0	0	0	0	0	0	0	0	0	0	0	0	0	0	0	0	4	164.5	41.0	110	121.5	174.5	197.5	199	0	2	2	4	65.8	11.3	49	54.3	70.0	73.0
U	0	1	0	0	0	0	1	0	0	0	0	0	0	0	0	0	0	0	0	0	0	5	125.6	21.5	111	112.5	116.0	143.5	163	0	5	0	5	61.6	16.5	42	46.0	60.0	78.0
V	0	0	0	0	0	0	0	0	0	0	0	0	0	0	0	0	0	0	0	0	3	5	154.6	32.3	126	126.5	150.0	185.0	204	0	4	1	5	74.4	21.8	42	53.0	83.0	91.5
W	1	0	1	0	0	0	0	0	0	0	0	0	0	0	0	0	0	0	0	0	0	3	143.7	14.2	134	134.0	137.0	160.0	160	0	3	0	3	91.3	15.1	74	74.0	98.0	102.0
X	1	0	0	0	0	0	0	0	0	0	0	0	0	1	0	0	0	0	0	0	0	2	131.0	35.4	106	106.0	131.0	156.0	156	0	2	0	2	80.0	5.7	76	76.0	80.0	84.0

Hospital Name	Heart rate (max)	Heart rate: 1-59	Heart rate: 60-99	Heart rate: ≥100	Respiratory rate (n)	Respiratory rate (mean)	Respiratory rate (SD)	Respiratory rate (min)	Respiratory rate (25percentile)	Respiratory rate (median)	Respiratory rate (75percentile)	Respiratory rate (max)	Respiratory rate: 0	Respiratory rate: 1–5	Respiratory rate: 6–9	Respiratory rate: > 29	Respiratory rate: 10–29	Serum Glucose (n)	Serum Glucose (mean)	Serum Glucose (SD)	Serum Glucose (min)	Serum Glucose (25percentile)	Serum Glucose (median)	Serum Glucose (75percentile)	SBP (max)	Serum glucose: <200	Serum glucose: ≥ 200	Mydriasis: Absent	Mydriasis: Present	AEDH: Absent	AEDH: Present	ASDH: Absent	ASDH: Present	ICH: Absent	ICH: Present	CC: Absent	CC: Present	tSAH: Absent	tSAH: Present
A	180	2	40	36	78	20.9	8.6	0	18.0	22.0	26.3	36	6	1	0	13	58	76	205.9	80.7	99	155.8	181.5	226.8	496	48	28	46	32	70	8	40	38	76	2	61	17	20	58
B	163	7	41	19	67	18.9	6.2	0	16.0	18.0	24.0	34	2	0	0	2	63	67	178.4	52.5	47	138.0	172.0	206.0	368	46	21	34	33	60	7	23	44	60	7	50	17	11	54
C	162	7	30	24	61	20.8	6.7	10	16.0	19.0	25.0	44	0	0	0	7	54	61	183.9	84.3	95	121.5	156.0	216.0	466	43	18	24	37	57	4	24	37	57	4	53	8	12	49
D	164	5	29	27	61	22.6	7.2	0	18.0	20.0	26.0	38	1	0	0	11	49	61	189.5	61.8	90	147.5	173.0	209.5	376	41	20	23	38	58	3	36	25	59	2	55	6	12	49
E	165	5	22	12	39	22.4	6.2	10	18.0	22.0	27.0	40	0	0	0	5	34	38	164.1	54.7	84	127.0	153.5	193.3	338	31	7	16	23	37	2	18	21	34	5	31	8	17	21
F	180	6	29	11	46	21.2	5.6	12	18.0	20.0	24.0	36	0	0	0	5	41	46	194.4	63.5	94	148.8	191.0	218.5	392	27	19	20	26	42	4	11	35	43	3	40	6	14	32
G	146	3	20	12	35	19.7	7.7	0	16.0	18.0	24.0	40	1	0	0	5	29	35	190.5	76.6	103	139.0	174.0	211.0	427	25	10	23	12	32	3	16	19	30	5	28	7	5	30
H	145	3	25	10	37	17.7	8.5	0	13.5	19.0	21.0	37	3	0	1	4	29	36	201.0	61.1	105	161.3	186.0	239.0	372	22	14	18	20	36	2	22	16	36	2	24	14	11	23
J	153	2	16	10	28	23.2	6.4	12	20.0	23.0	25.0	40	0	0	0	5	23	28	198.9	59.0	96	164.3	197.0	234.5	337	15	13	12	16	26	2	16	12	28	0	27	1	7	21
K	155	0	21	8	19	18.6	8.9	0	15.0	20.0	22.0	36	2	0	0	2	15	29	169.1	66.2	46	117.5	153.0	231.0	316	19	10	9	20	26	3	17	12	23	6	22	7	10	19
L	164	1	16	8	25	18.4	4.4	10	15.0	18.0	22.0	26	0	0	0	0	25	25	191.0	55.6	72	158.0	177.0	235.5	293	16	9	12	13	25	0	10	15	23	2	21	4	2	23
M	124	2	15	5	22	23.1	7.1	0	20.8	23.5	28.0	37	1	0	0	2	19	22	171.8	55.4	104	133.3	159.5	191.5	308	17	5	17	5	18	4	11	11	22	0	20	2	5	17
N	150	1	16	9	26	23.2	6.7	0	20.0	24.0	25.8	36	1	0	0	5	20	26	206.2	59.8	138	162.5	188.0	245.3	383	16	10	9	16	25	1	12	14	26	0	20	6	7	19
P	100	4	17	1	22	23.6	5.0	14	20.0	24.0	24.3	36	0	0	0	3	19	22	186.2	53.9	106	165.5	185.0	200.8	387	16	6	8	14	17	5	0	22	21	1	13	9	8	14
Q	131	0	8	2	1	17.0		17	17.0	17.0	17.0	17	0	0	0	0	1	9	170.9	67.1	97	115.0	154.0	235.5	285	6	3	7	3	8	2	2	8	9	1	9	1	2	8
R	134	2	7	5	14	22.4	9.5	10	14.3	19.5	29.3	44	0	0	0	3	11	10	195.2	61.7	122	156.3	190.0	212.0	346	7	3	9	6	13	2	11	4	15	0	14	1	8	7
S	152	2	10	2	12	21.0	6.2	10	18.0	19.5	28.0	30	0	0	0	1	11	14	183.4	71.5	108	112.8	167.0	269.0	315	9	5	5	9	13	1	5	9	14	0	11	3	3	11
T	74	1	3	0	4	18.5	1.9	16	16.5	19.0	20.0	20	0	0	0	0	4	4	198.8	16.4	185	185.8	194.5	216.0	221	2	2	0	4	3	1	3	1	3	1	4	0	3	1
U	82	2	3	0	5	16.0	5.1	10	11.0	16.0	21.0	22	0	0	0	0	5	5	174.2	47.7	131	138.5	166.0	214.0	254	4	1	3	2	4	1	3	2	4	1	4	1	0	5
V	98	1	4	0	0								0	0	0	0	0	5	171.4	24.3	144	147.0	179.0	192.0	203	4	1	1	4	5	0	1	4	5	0	3	2	1	4
W	102	0	2	1	3	24.0	6.0	18	18.0	24.0	30.0	30	0	0	0	1	2	3	177.7	46.6	124	124.0	201.0	208.0	208	1	2	2	1	3	0	2	1	3	0	3	0	1	2
X	84	0	2	0	2	13.0	1.4	12	12.0	13.0	14.0	14	0	0	0	0	2	2	209.0	5.7	205	205.0	209.0	213.0	213	0	2	1	1	2	0	2	0	2	0	2	0	0	2
